# Mobilization and Activation of the Innate Immune Response to Dengue Virus

**DOI:** 10.3389/fcimb.2020.574417

**Published:** 2020-11-03

**Authors:** Christine A. King, Adam D. Wegman, Timothy P. Endy

**Affiliations:** Department of Microbiology and Immunology, State University of New York (SUNY) Upstate Medical University, Syracuse, NY, United States

**Keywords:** dengue, innate immunity, macrophages, mast cells, interferon, inflammation, pathogenesis, clinical symptoms

## Abstract

Dengue virus is an important human pathogen, infecting an estimated 400 million individuals per year and causing symptomatic disease in a subset of approximately 100 million. Much of the effort to date describing the host response to dengue has focused on the adaptive immune response, in part because of the well-established roles of antibody-dependent enhancement and T cell original sin as drivers of severe dengue upon heterotypic secondary infection. However, the innate immune system is a crucial factor in the host response to dengue, as it both governs the fate and vigor of the adaptive immune response, and mediates the acute inflammatory response in tissues. In this review, we discuss the innate inflammatory response to dengue infection, focusing on the role of evolutionarily conserved innate immune cells, their effector functions, and clinical course.

## Introduction

### Dengue Virus and Clinical Spectra

Dengue virus (DENV) is an arbovirus transmitted by the mosquito vectors *Aedes aegypti* and, to a lesser extent, *Aedes albopictus* (Scott and Morrison, 2010). Dengue virus belongs to the family Flaviviridae and is a single stranded, positive sense, enveloped, RNA virus. The genome is approximately 11 kb and encodes 10 proteins. Upon infection the viral genome is delivered to the cytoplasm and translated into one long polyprotein that is then cleaved by both host and viral specific proteases to yield 10 individual proteins. Three are structural proteins (envelope, core, and membrane) and seven are non-structural (NS) proteins (NS1, NS2a and NS2b, NS3, NS4a and NS4b, and NS5). Dengue is endemic in tropical and subtropical regions of the world where 2.5 billion people are at risk for infection. With approximately 400 million infections annually (WHO, 2009; Bhatt et al., 2013), dengue disease is a serious public health threat with no specific treatments currently available. There are currently four circulating serotypes (DENV-1 to 4) that exhibit up to 70% sequence homology (Blok, 1985; Green and Rothman, 2006). All four serotypes can cause a spectrum of disease with manifestations ranging from a subclinical infection to a mild febrile illness termed dengue fever (DF). In a subset of infections, severe hemorrhagic manifestations or shock syndrome known as dengue hemorrhagic fever (DHF) and dengue shock syndrome (DSS) (WHO, 2009) can develop. While the majority of patients develop only mild symptoms and recover after defervescence, approximately 5% develop life threating vascular dysfunction (Gubler, 1998; Halstead, 2007). The pathogenesis of severe dengue disease has been the focus of countless studies, and some progress in understanding disease associations and mechanisms has been made. What is known is that severe dengue disease most often occurs during a secondary DENV infection with a heterologous serotype (Halstead, 1994; Gubler, 1998; Halstead, 2007). This phenomenon is thought to involve antibody-dependent enhancement (ADE) which is characterized by the enhanced infection of target cells *via* Fcγ receptor bearing cell-mediated internalization of IgG coated virus. The hypothesis suggests that cross-reactive antibodies that bind virus are not neutralizing, or are at sub-neutralizing concentrations, (Halstead and O’Rourke, 1977a) thereby facilitating infection, rather than preventing. Several *in vivo* lines of evidence support this hypothesis (Halstead and O’Rourke, 1977a; Halstead and O’Rourke, 1977b; Zellweger et al., 2010). Both *in vitro* experiments in K562 cells and *in vivo* experiments with juvenile rhesus macaques demonstrated that ADE led to increased titers, with up to a 1000-fold increase *in vitro* and a 100-fold increase *in vivo* (Goncalvez et al., 2007). Higher levels of viremia are correlated with increased dengue disease severity in humans (Vaughn et al., 2000). There is also evidence that immature DENV virions are rendered highly infectious by anti-prM antibodies (Goncalvez et al., 2007; Rodenhuis-Zybert et al., 2010). Moreover, F_c_ receptor signaling during immune complex binding is not restricted to the internalization event; other signaling includes suppression of IFN-gamma transcription and translation, increased synthesis of IL-6, and downregulation of IRF-1 and STAT1 [reviewed in (Halstead et al., 2010)]. F_c_ receptor engagement also reportedly downregulates RIG-I/MDA5 signaling and decreases production of type I interferon (Chareonsirisuthigul et al., 2007).

The host-specific immune responses to DENV likely play a large role in the pathophysiology of disease and subsequent clinical manifestation of dengue infection. Dengue disease is a complex viral-host interaction with not only cross reactive antibody and T cell immunity as important determinants of severity (Mongkolsapaya et al., 2003; Friberg et al., 2011; Midgley et al., 2011), but also host genetics including polymorphisms in the TNF and lyphotoxin receptor (Fernandez-Mestre et al., 2004; Vejbaesya et al., 2009)and MHC class I alleles (Stephens et al., 2002; Zompi and Harris, 2012). These studies have found that several polymorphisms in these alleles are associated with more severe dengue disease, while others, particularly in the MHC alleles can be associated with less severe disease. For example, HLA A*0203 is associated with less severe dengue fever, while HLA*0207 is associated with more severe DHF and DSS in secondary infection. By contrast, HLA B44, B62, B76, and B77 are associated with protection against developing clinical disease after secondary dengue infection (Stephens et al., 2002). Virus virulence factors are also associated with severity of disease including the sequence of specific serotypes causing infection (Halstead et al., 1983; Morens and Halstead, 1987; Halstead, 1998). The wide range in clinical presentation is likely the result of the interaction of many variables, both virus and host. Clinical presentation can include severe headache; retro-orbital eye pain; muscle, joint, and bone pain; nausea; vomiting, macular or maculopapular rash; a positive tourniquet test; or other hemorrhagic manifestations such as petechia, ecchymosis, purpura, epistaxis, bleeding gums, and hematuria. Severity is associated with warning signs including abdominal pain or tenderness, persistent vomiting, clinical fluid accumulation, mucosal bleeding, lethargy, restlessness, and liver enlargement (Deen et al., 2006). The hallmark for DHF and DSS is plasma leakage characterized by endothelial damage and leakage of intravascular plasma to the extravascular space (Srikiatkhachorn, 2009; Rothman, 2010).

In this review, we focus on the innate inflammatory responses to DENV infection by innate immune cells, their effector functions and clinical course.

## Models for Dengue Virus Immune Response

DENV, and accompanying clinical disease, are almost entirely restricted to humans and non-human primates, and the latter are largely asymptomatic. Few small animal models exist (Yauch and Shresta, 2008; Zellweger and Shresta, 2014), as rodent cells are generally not permissive to DENV infection, and each presents significant challenges for use and extrapolation to humans. To circumvent the issue of infectability, mouse models are genetically modified to be permissive to virus infection, most often by targeting the IFN system, and by adapting the virus. As such, immunocompromised mice are a common model for studying DENV pathogenesis and immunity. The most widely used model is the AG129 IFNα/β/γ receptor knockout mouse that when infected with a mouse-adapted DENV strain, recapitulates aspects of severe dengue disease (Yauch et al., 2009; Watanabe et al., 2012) including vascular leak and ADE (Shresta et al., 2006; Balsitis et al., 2010). The NOD/SCID/IL-2RγKO mice engrafted with human CD34+ stem cells develop symptoms of mild DF (Bente et al., 2005; Mota and Rico-Hesse, 2011), while the RAG-hu mouse model develops fever only (Kuruvilla et al., 2007). These models are not ideal in which to study the immunopathogenesis of a human-constrained virus. Dengue does not naturally infect rodents. Moreover, the use of these models requires adaptation of human dengue strains in order to establish any infection. This, coupled with the required immune knockouts to generate infection and disease, makes it difficult to apply knowledge gained in immune deficient murine systems to the events occurring in human hosts with intact immune systems. Much of the pathogenesis of dengue disease is thought to be due to activation of the immune system, and these models do not recapitulate a fully functioning immune system. However, immunocompetent C57BL/6j and BALB/c Mice infected IP with a mouse adapted passaged clinical isolate of DENV 3 exhibit severe disease and die by day 6–7 post-infection, recapitulating many of the observed clinical signs of severe dengue including thrombocytopenia, decrease in systolic blood pressure, increased liver enzymes, and viremia (Goncalves et al., 2012).

These animal models are not sufficient to test antivirals, understand mechanisms of clinical symptoms, or select vaccine candidates. Limited knowledge of the range and complexity of the immune response generated in humans makes it particularly challenging to design an effective vaccine. Illustrating this, the only available vaccine, licensed in 2019, was shown to sensitize some seronegative recipients to more severe dengue disease upon infection with wild-type virus (Biswal et al., 2019). These limitations arise, in part, from an incomplete understanding of the human immune response to DENV, which is essential to exploit when designing a vaccine. Importantly, the early innate immune response governs the fate and vigor of the subsequent adaptive immune response (Fearon and Locksley, 1996), which confers the long-term protection desired from a vaccine. Compounding this problem, epidemiological studies in dengue-endemic areas are limited in several ways: they cannot control for many important factors in their subjects (e.g. prior flavivirus exposure), and are limited to enrolling virologically-confirmed cases of dengue who present with symptoms. These limitations leave a substantial gap in our knowledge of the early innate immune response to DENV.

Controlled human infection models (CHIMs) have been used successfully and safely for a number of human pathogens including cholera, influenza, malaria, typhoid and other enteric pathogens. CHIMs for DENV challenge studies have been historically and currently used as a safe means to test vaccine product viability (Thomas, 2013; Endy, 2014). The Walter Reed Army Institute of Research (WRAIR) initiated the development of a DENV human infection model (DHIM) in 2001 using previous DENV vaccine viruses that were found to be too reactogenic for a vaccine, but safe in human trials and potential candidates for a challenge virus (Lyons, 2014). The first study was conducted in 2001 in 15 volunteers. Two subjects received DENV-1, three subjects received DENV-2, three subjects received DENV-3, four subjects received DENV-4, and three subjects received placebo. DENV-1 strain 45AZ5 was administered subcutaneously (SC) at a dose of 0.5 ml containing 1.6 x 10^4^ PFU (Mammen et al., 2014). Challenge resulted in a mild dengue fever-like illness with fever, chills, myalgias, arthralgias, headache, eye pain, malaise, anorexia, backache, abdominal pain, pruritus, photosensitivity, lymphadenopathy, and loose stools. Also observed were a morbilliform truncal rash, lymphadenopathy, leukopenia, neutropenia, and small perihepatic effusion. A follow-up study was performed in 2008 using both previously vaccinated and non-vaccinated volunteers. In those who received DENV-1, all 5 subjects previously vaccinated with a tetravalent live-attenuated dengue vaccine were protected against DENV-1 virus challenge (Sun et al., 2013). In volunteers who did not receive the vaccine, dengue fever like symptoms and laboratory findings occurred. In both trials, volunteers had resolution of their symptoms and viremia without serious adverse events. We recently completed a phase I study of 12 healthy adult volunteers using a challenge virus, DENV-1-LVHC, strain 45AZ5 (Endy et al., 2020). All subjects developed neutralizing antibody to DENV-1, and 11 of the 12 developed viremia with peak viral loads similar to wild-type DENV infection. There were no serious consequences to infection and all recovered without problems. The DHIM offers a platform in which to test the viability of candidate vaccines and therapeutics. Equally important, it offers a reproducible model in which to study the viral-host interactions and the innate and adaptive immune response to DENV infection.

## Innate Immune System

The ability to respond to and orchestrate effective defenses against invading pathogens is a key element of survival. The human immune system achieves this by effectively controlling dangerous pathogens and ignoring the rest. Composed of two arms, the innate arm and the adaptive arm, The innate arm is activated in response to both injury and infection; the adaptive arm is recruited and activated in response to innate immune activation and direction, is specific, and is tailored for individual pathogens [reviewed in (Dorothy and Lewis)].

Evolutionarily conserved, the innate system harnesses the power of molecular and danger patterns to activate a highly specific inflammatory response aimed at alerting and mobilizing immune cells, with the ultimate goal being to clear the invading pathogen and/or repair the damage that initiated the response [reviewed in (Janeway and Medzhitov, 2002; Barton, 2008)]. By contrast, the adaptive system is highly specific, with recognition mediated by and activation tailored to antigen encountered, and requires appropriate secondary signals. The effectors of the adaptive immune system include T cell and B cells. The innate system is more complex [reviewed in (Douglas and McDonald, 2019)]; it is composed of monocytes, macrophages, neutrophils, mast cells, basophils, eosinophils, NK cells and ILCs. Many of these are granulocytes that store preformed mediators, including specific inflammatory proteases and cytokines, for immediate release. These granulocytes also *de novo* synthesize a plethora of highly inflammatory cytokines, chemokines, and Cox-2- and 5-lipoxygenase-derived lipid mediators (Alvarez et al., 2010) that are necessary to induce and direct an appropriate adaptive immune response. Activation of tissue resident macrophages and mast cells at the area of wound or pathogen entry initiates a cascade of events aimed ultimately at healing and pathogen clearance, often with the help of the adaptive arm. The direct effect of these inflammatory mediators is to modify endothelial tight junctions and adhesion molecules to allow for influx of immune cells from the circulation, to recruit other innate immune effectors to the site, and to activate nearby tissue resident cells to mount a response against the insult. Upon activation of mast cells, dendritic cells are activated and directed to egress from the site, homing to the draining lymph node to activate the adaptive arm of the immune system (Jawdat et al., 2006; Suto et al., 2006; Dudeck et al., 2019). The evolutionary importance of these tissue resident cells is underscored by the finding that both phagocytes (macrophages) and granulocytes (mast cells) are highly conserved, found in a range of Kingdoms and species including invertebrates and primitive chordates (Rhodes et al., 1982; Crivellato and Ribatti, 2010; Wittamer et al., 2011). It is these tissue resident cells that initiate the acute inflammation required to alert and instruct both the innate and adaptive arms of the immune system to the danger/pathogen, allowing for effective mobilization of the appropriate cellular compartment to the site.

## Professional Phagocytes of the Skin: Macrophages, DCs, Langerhans Cells

There are several valid ways of subcategorizing phagocytic immune cell populations. The macrophage/DC/LC field is currently undergoing a shift away from defining these cell types and subtypes by their functional/phenotypic properties, and towards defining the populations by ontogeny (developmental lineage) and transcription factor expression/transcriptome profiles. In consequence, LCs/DCs and subsets and macrophages have not always been defined identically. Where appropriate, we have denoted specific subsets with their definitive CD antigens in order to facilitate comparisons between human and mouse data, as well as to aid in comparing studies where immune cell subtypes may not have been defined identically.

Dendritic cells are phagocytic cells derived from CD34^+^ hematopoietic cell precursors that give rise to both myeloid and lymphoid precursors. Dendritic cells are considered the most efficient antigen-presenting cell whose canonical function is to activate naïve T cells. In this capacity, dendritic cells capture, process and present antigens in MHC Class II to CD4 + T cells [reviewed in (Banchereau et al., 2000)]. They express high levels of MHC Class II and CD11c in addition to a range of other surface markers that identify distinct subtypes. As tissue-resident cells, they are relatively short-lived, and require replenishment from bone marrow-derived precursors in order to maintain their numbers in peripheral organs [reviewed in (Doebel et al., 2017; Collin and Bigley, 2018; Macri et al., 2018)]. Dendritic cells exist in two functional states, immature and mature [reviewed in (Worbs et al., 2017)]. In tissues they are immature, with a decreased ability to induce naïve lymphocyte effector responses but with a robust ability to capture antigen. Dendritic cells undergo a maturation program both as a result of macrophage and mast cell activation-derived signals and also by sensing of danger- or pathogen-associated molecular patterns. Maturation activates dendritic cells to migrate to secondary lymphoid organs where they function to present antigen to T cells and promote the initiation of adaptive immunity [reviewed in (Patente et al., 2019)]. Langerhans cells are epidermal innate immune cells of myeloid origin. They bear some functional similarities to dendritic cells, including the capacity to migrate to lymph nodes and stimulate T cells. However, classified according to developmental origin (ontogeny), they are considered a specialized subset of macrophages: they arise from embryonic precursors rather than bone marrow. Langerhans cells are long-lived in tissue and self-maintain their population without replenishment (Doebel et al., 2017).

## The Infection Event: Anatomy and Mosquito Factors

**Figure 1:**
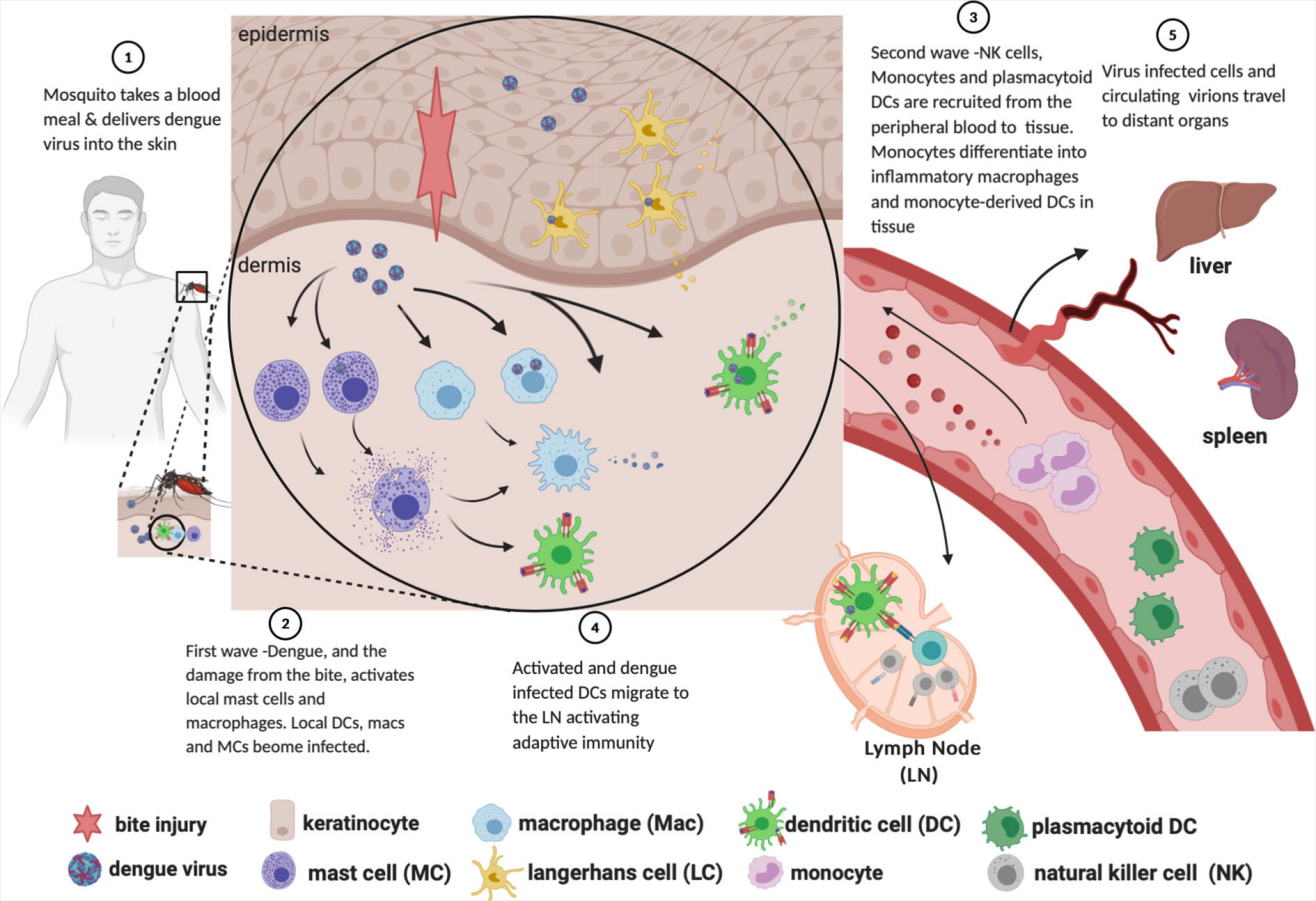
Path of dengue infection from mosquito bite to dissemination to organs. Created with Biorender.com.

DENV is introduced into the skin *via* saliva deposition as a mosquito takes a blood meal (**Figure 1**, Step 1). The mosquito proboscis must necessarily penetrate through the epidermis and into the dermis in order to access the capillary beds, though the specific distribution of virions introduced during the feeding event is unclear. Some sources suggest that DENV is directly injected into the dermis instead of the epidermis (Begum et al., 2019); others assert that the virus is introduced into both layers (Garcia et al., 2017; Rathore and St John, 2018); and still others claim that virions are introduced directly into the bloodstream, with “spillover” into both the dermis and epidermis (Martina et al., 2009). As discussed below, susceptible and permissive cells from both layers are infected soon after inoculation, supporting the notion that DENV is not restricted to a specific layer of the skin after the inoculation event.

In addition to viral particles, feeding mosquitos also inject several salivary proteins. These, in addition to local anticoagulant activity, have been shown to facilitate the establishment and virulence of several flaviviruses. Inoculation of C57BL/6 mice with West Nile Virus with infected *Culex tarsalis* mosquitoes results in increased viremia 24 and 48 h.p.i. compared to needle injection of a comparable inoculum (Styer et al., 2011). While the precise mechanism was not determined, possibilities include local immunomodulation at the bite site increasing WNV tropism, and/or leukocyte infiltration increasing the number of WNV-susceptible cells at the bite site. C57BL/6 mice passively immunized against the *Aedes aegypti* salivary factor NeSt1 showed reduced early ZIKV replication, reduced macrophage infiltration at the bite site, and increased 30-day survival; this is possibly due to reduced levels of pro-IL-1β and CXCL2 at the bite site (Hastings et al., 2019). Humanized (NSG) mice infected by *A. aegypti* mosquitoes exhibit prolonged DENV viremia; moreover, a mosquito bite containing no virus induced an innate immune cytokine response comprising TNF, IL-4, and IL-10, underscoring that mosquito saliva is itself immunomodulatory in the absence of virus (Cox et al., 2012). From a mechanistic standpoint, the *A. aegypti* salivary protein CLIPA3 has been implicated as a specific factor contributing to increased DENV infectivity, facilitating attachment of viral particles to cell surface receptors and cell migration *via* digestion of extracellular matrix (Conway et al., 2014). These experiments have not elaborated the range of mosquito salivary factors involved, but point to the initial inoculation conditions, and thus the innate immune response of the skin, as an important variable influencing the course of infection. It should be noted that much of the dengue field involving mouse or non-human primates, as well as the current human infection models, do not account for this variable when inoculating with DENV.

Once deposited, DENV is capable of infecting and replicating in keratinocytes and fibroblasts (Garcia et al., 2017) as well as the several populations of professional skin-resident phagocytic cells: Langerhans cells, dendritic cells, and macrophages (Wu et al., 2000; Cerny et al., 2014; Schmid and Harris, 2014; Garcia et al., 2017; Rathore and St John, 2018). A specific entry receptor or family of receptors for DENV, and thus the range of cellular tropism, has not yet been established. Aside from antibody-dependent enhancement, native cell-surface candidates for DENV internalization include L-SIGN, DC-SIGN, C-type lectins, the mannose receptor, glycosaminoglycans such as heparin sulfate, TIM-1, TAM, CD14, and CD300a (Crill and Roehrig, 2001; Cruz-Oliveira et al., 2015; Ngono and Shresta, 2018). The DENV E protein structural domain III is the most likely candidate for binding the cellular entry receptor (Crill and Roehrig, 2001; Cruz-Oliveira et al., 2015) but, owing in part to the range of putative entry receptors, the exact binding motif(s) remains unknown.

## FIRST WAVE: Tissue-Resident Phagocytes and Keratinocytes Productively Infected

Work primarily done in C57BL/6 mice lacking the interferon alpha receptor (*Ifnar ^-/-^*) (Schmid and Harris, 2014) and thoroughly reviewed in (Rathore and St John, 2018), demonstrated that there are two “waves” of cells infected with DENV following inoculation. In the first wave (infection by the initial inoculum), mast cells, macrophages, CD103+ DCs, and Ly6C-CD11b+ DCs—analogous to human CD141+ conventional (myeloid) dendritic cell subtypes 1 and 2, respectively (St John et al., 2011; Collin and Bigley, 2018)—are infected with DENV (**Figure 1**, Step 2). Other studies employing non-human primates and cadaveric human skin explants, in addition to the above studies in *Ifnar ^-/-^* mice, have shown that Langerhans cells are among those initially infected (Taweechaisupapong et al., 1996a; Taweechaisupapong et al., 1996b; Wu et al., 2000).

While the infection of LCs, DCs, and macrophages is well known, there is some disagreement as to which layer of the skin carries the highest burden of infection. Studies done in intradermally inoculated *Ifnar ^-/-^* mice show up to 100-fold more DENV-infected cells in the dermis as compared to the epidermis at 72 h post-inoculation (Schmid and Harris, 2014; Schmid et al., 2014). These flow cytometry data were gated on CD45+ cells taken from the skin of the mice, thereby excluding from consideration keratinocytes, which do not express CD45. By contrast, subsequent work by Duangkhae and colleagues demonstrated that keratinocytes alone accounted for up to 60% of DENV-infected cells in a human skin explant model, and that the epidermis contained approximately six-fold more DENV-infected cells than the dermis at 48 hpi (Duangkhae et al., 2018). While a consensus of infection burden between the skin layers has yet to be reached, the available evidence is generally clear that keratinocytes and skin-resident phagocytes are infected after introduction of virus and prior to development of viremia. These infected cells, while mounting an innate immune response against the virus, simultaneously create a milieu conducive to propagating the number of infected cells. For example, administration of neutralizing Ab to IL-1β into skin at 2 h.p.i. decreased the burden of infected dermal cells by 65% (Duangkhae et al., 2018). These data illustrate the feed-forward effect that leads to the second wave of infection.

### General Cellular Host Response to DENV and Clinical Consequences

During viral replication, intracellular DENV ssRNA and dsRNA, intermediates of viral genome replication, are recognized as pathogen-associated molecular patterns (PAMPs) by host cell pattern recognition receptors (PRRs) including MDA5, RIG-I, TLR3, and TLR7 (Pichyangkul et al., 2003; Rothman et al., 2003; Morrison and García-Sastre, 2014; Cumberworth et al., 2017; Ngono and Shresta, 2018; Uno and Ross, 2018; Tremblay et al., 2019). Engagement of these receptors triggers an antiviral response, which is initially characterized by the induction of the type 1 interferon (IFN) response. Briefly, intracellular signaling pathways downstream of the above PAMP receptors culminate in activation of the master innate immune transcription factors IRF3, IRF7, and NF-κB, which direct the transcription and secretion of IFN-α and IFN-β. These proteins in turn act in both an autocrine and paracrine fashion to produce a general antiviral intracellular milieu hostile to virion production (Rothman et al., 2003; Morrison and García-Sastre, 2014; Cumberworth et al., 2017; Uno and Ross, 2018; Tremblay et al., 2019). Thus, the initial stages of DENV infection proceed in a positive-feedback cycle of an increasing burden of cells infected, as well as an increasing capacity to mount a type 1 interferon response.

The cellular immune response at large—not limited to type 1 IFN secretion—as well as an increasing burden of infected cells contributes to the subsequent clinical manifestations of dengue. In one human subject who received a tetravalent live DENV vaccine, infected dendritic cells and Langerhans cells were found in a cutaneous rash distant from the injection site (Wu et al., 2000). The investigators performed immunohistochemical staining for DENV glycoprotein from a skin biopsy and confirmed the presence of infected DCs and LCs, indicating an association between presence of DENV and clinical symptoms manifesting in the skin. Production of the pyrogen IL-1β begins with the first wave of infected cells; additionally, production of TNF, responsible for pyrexia, myalgia and appetite suppression, could begin at the stage of skin infection as well (Schaeffer et al., 2015). More broadly, type 1 interferon contributes to a gamut of clinical symptoms, the range of which is clearly delineated during administration of exogenous interferon in a therapeutic setting: fever, chills, headache, and fatigue (Todd and Goa, 1992; Sleijfer et al., 2005; Owen et al., 2013; Torkildsen et al., 2016).

### DENV: Subversion of the Interferon Response

Though the IFN response is critical to dampen DENV replication (Diamond et al., 2000), it is insufficient to completely suppress production of infectious DENV. Indeed, historical studies in humans [reviewed in (Snow et al., 2014)] as well as human data from our lab (Endy et al., 2020) suggest that not every DENV inoculation results in detectable viremia. However, the typical finding of viremia in symptomatic cases, and the presence of DENV antigen in several cell types beyond the site of infection (Begum et al., 2019), indicates that infection is not routinely confined to the skin. This is likely due to subversion of the human interferon response by DENV. At a tissue level, data from human skin explants show transient, rather than sustained, production of IFN-α by skin cells after DENV infection, with peak levels occurring less than 12 h.p.i. (Duangkhae et al., 2018). At the cellular level, several DENV nonstructural (NS) proteins suppress both induction and downstream signaling of interferon. NS2a, NS4a, and NS4b block TBK1 phosphorylation, preventing the transcription of IFN-β (Dalrymple et al., 2015). NS2b targets cGAS for degradation, and NS2b/3 cleaves the intracellular DNA sensor STING, representing multiple points of disruption to the cGAS-STING pathway which would otherwise induce interferon (Lee et al., 2018). NS5 2’-O-methylates the 5’ cap of the DENV genome, which prevents detection by RIG-I (Lee et al., 2018). NS4a binds to the CARD-like and transmembrane domains of MAVS, preventing binding of RIG-I and therefore activation of IRF3 and consequent interferon induction (He et al., 2016).

**Figure 2:**
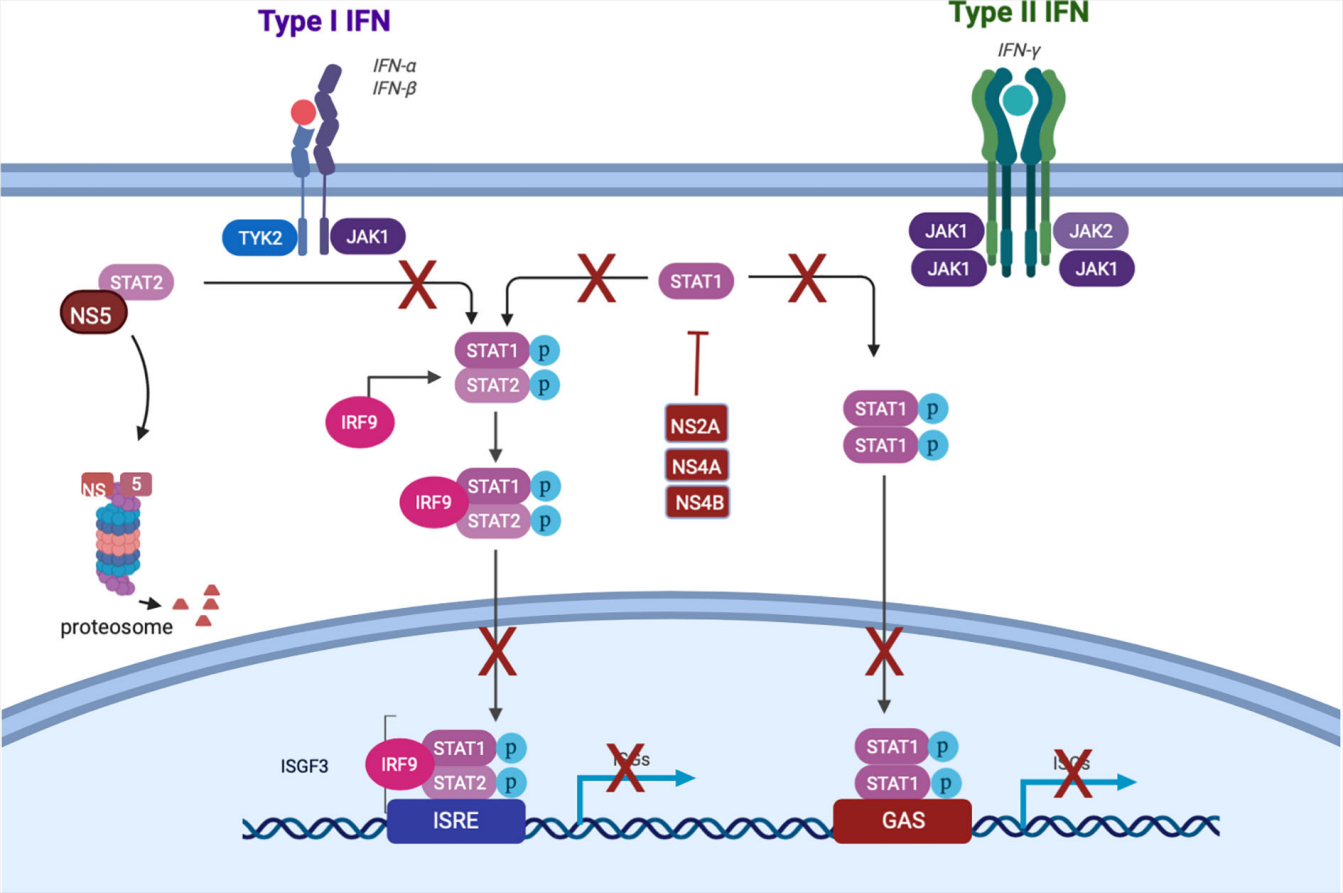
Dengue subversion of interferon signaling. Created with Biorender.com.

Not all DENV antagonism of the IFN response occurs upstream of interferon production itself. This is important when considering that pDCs (discussed below in the *Second Wave* section) produce IFN without being infected. Thus, DENV also has the capability to disrupt the signaling downstream of the IFNAR: NS2a, NS4a, and NS4b inhibit STAT1 phosphorylation, and NS5 mediates proteasomal degradation of STAT2 (**Figure 2**) (Morrison and García-Sastre, 2014; Tremblay et al., 2019). DENV has genome size constraints and limited coding capacity expressing 10 proteins, seven of which are non-structural; five of those function to target an aspect of the IFN system. This intense focus of interfering with the IFN system underscores how important this system is in controlling DENV infection. Together, these mechanisms suppress IFN activation and downstream signaling, enabling DENV to replicate locally and to produce virions that infect cells beyond the bite site.

### Tissue Resident Mast Cells Are Activated During Dengue Infection

Mast cells (MCs) are long-lived, CD34+ (Maaninka et al., 2013) tissue-resident innate immune inflammatory cells that reside in almost every tissue and organ, including in high levels in the skin (Galli et al., 2005a). In skin, mast cells are positioned in the dermis, adjacent to microvessels, where they serve as sentinels and sense blood borne and tissue localized pathogens to direct immune cell recruitment to the area. The perivascular location also facilitates MC-driven modulation of EC function (Klein et al., 1989; Brown et al., 2011; Kunder et al., 2011), and several mast cell specific -mediators have been shown to promote EC activation (Compton et al., 1998; Frossi et al., 2004), a response necessary for immune cell recruitment. MCs recognize and become activated by a wide range of stimuli, including foreign pathogens [reviewed in (Marshall and Bienenstock, 1994; Marshall and Jawdat, 2004; Abraham and St John, 2010)] to drive a rapid, pro-inflammatory response (Galli et al., 2005b; Metz et al., 2007; Metz and Maurer, 2007) involving protease release, eicosanoid synthesis and release, and *de novo* synthesis of cytokines, chemokines and growth factors, including Type 1 IFNs.

Mast cell activation *in vivo* in tissue and *in vitro* by DENV induces degranulation with release of granule contents (**Figure 1**, Step 2) (St John et al., 2011; Troupin et al., 2016) that stimulates activation of the endothelium (Brown et al., 2011; Kunder et al., 2011) and likely contributes to the clinical rash often seen during the clinical course of infection (Nakamura et al., 2009; Mishra et al., 2018). Activated mast cells synthesize and release CXCL2, a potent chemotactic factor for neutrophils, and have been shown to promote neutrophil activation and recruitment from the periphery (Zhang et al., 1992; Abraham and Malaviya, 1997). *In vivo* mouse studies and *in vitro* human models have demonstrated mast cells are permissive to dengue virus infection *via* ADE (King et al., 2000). One of the key responses is *de novo* synthesis of pro-inflammatory cytokines TNF (Brown et al., 2011) and IL-1β (King et al., 2000), and the chemokines CCL3, CCL4 and CCL5 (King et al., 2002), CXCL12, and CXCL1 (St John et al., 2011). Mast cells respond to DENV antibody-mediated infection with a robust IFN expression by 12 h post-infection that is maintained up to 72 h (Brown et al., 2011). *Via* degranulation, release of Type 1 IFNs, and chemotactic factors, MCs initiate local acute immune activation, providing the required signals for activation and chemotactic recruitment of circulating monocytes, local tissue macrophages, NK cells and neutrophils. Subsequent work with MC-deficient mice demonstrated the importance of mast cell-driven recruitment of natural killer and natural killer T cells into the infected skin. In this model, mast cells were critical for containing DENV *in vivo*, and without which there was increased viral burden within draining lymph nodes after subcutaneous infection compared to MC-sufficient mice (St John et al., 2011).

Human studies have demonstrated that mast cells exhibit an extensive activated phenotype with degranulation in the rash of infected subjects (Asher AN et al., 2002) and dengue disease severity associated with levels of mast cell granule stored mediators including chymase (Avirutnan and Matangkasombut, 2013; Tissera et al., 2017), histamine (Tuchinda et al., 1977), VEGF (Furuta et al., 2012), and tryptase (Rathore et al., 2019). Disease severity is also associated with IgE, a classical mast cell activating antibody (Koraka et al., 2003). Together, these data strongly suggest a role for dengue-driven mast cell mediator release in clinical symptoms of dengue disease. In addition, a large histological study of DHF patients in Thailand determined that mast cells in connective tissue showed evidence of activation including swelling, vacuolation of the cytoplasm, and loss of granule integrity (Bhamarapravati et al., 1967). Mast cell activation [reviewed in (Valent, 2013; Afrin et al., 2017)] is known to mediate several of the common clinical symptoms associated with dengue patients including rash, diarrhea, vomiting, headache, eye pain/inflammation and muscle pain. Given the potency of mast cell activation and the resultant inflammation and clinical symptoms, targeting vasoactive mast cell mediators such as histamine or tryptase may have a significant impact of clinical course. In a small study in Pakistan, treatment with anti-histamines and steroids dramatically reduced dengue symptoms, shortening duration to 3–5 days, as compared to the other treatment groups of 7–10 days (Siddique et al., 2008). An older study on 24 patients in the Armed Forces found even low dose targeting of histamine reduced the duration of clinical symptoms, suggesting that interfering with mast cell mediators or activation may have a role in treating dengue disease (Hoffman et al., 1954). More recently, a randomized clinical trial in 200 dengue patients demonstrated that a single daily dose of 10 mg montelukast, a cysteinyl leukotriene inhibitor that blocks leukotriene C4, D4, and E4 eicosanoids from binding their cognate cysteinyl leukotriene receptor 1, significantly reduced incidence of severe dengue shock syndrome by 71%, as compared to the control group (Ahmad et al., 2018). Montelukast is a standard cysteinyl leukotriene receptor inhibitor given to asthma patients to help control the mast cell-derived inflammatory leukotriene responses to reduce airway inflammation. Given that dengue virus induces degranulation of tissue resident mast cells, that rash exists at sites distant to inoculation, and that in our human infection model rash is present both before and after resolution of DENV viremia (Endy et al., 2020), we suggest that perhaps DENV is present at high levels in skin sites throughout disease. In this context, rash would then be a direct clinical sign of DENV virus replication, as has been observed in other severe viral infections, including measles and pox virus skin lesions.

## SECOND WAVE—Monocyte-Derived Macrophages and DCs Recruited and Infected; Plasmacytoid DCs Recruited and Activated

Following the first wave of cells infected in the skin, local myeloid cells, monocyte-derived macrophages, monocyte-derived dendritic cells (moDCs, Ly6C+CD11b+), and circulating plasmacytoid dendritic cells (pDCs, CD123+) are recruited into the skin *via* chemokine signals (**Figure 1**, Step 4). These signals are secreted from tissue-resident macrophages, mast cells and other initially infected resident cells. Myeloid cells are recruited to the site of inflammation *via* CCL2, IL-1β and CCL20 (Dieu-Nosjean et al., 2000; Rider et al., 2011; Schmid and Harris, 2014; Schmid et al., 2014; Duangkhae et al., 2018); monocyte extravasation from peripheral blood is facilitated by CCL1 and CCL5 (Shi and Pamer, 2011); and pDC recruitment to inflamed skin is facilitated by CCL2, among others (Swiecki and Colonna, 2015).

Upon arrival, moDCs and macrophages are productively infected with DENV (Schmid and Harris, 2014), with some *in vitro* evidence suggesting that moDCs are up to ten times more permissive to infection than either monocytes or macrophages (Navarro-Sanchez et al., 2003). Of interest, dengue virions shed by mosquito cells have a different tropism for human cells than those shed by human DCs, with the former able to bind both DC-SIGN and L-SIGN, and the latter only able to bind L-SIGN (Dejnirattisai et al., 2011). This factor, which is not controlled for in many experimental models, complicates the analysis of the kinetics and infected cell burden of the second wave. pDCs, on the other hand, are not productively infected themselves; they are instead activated by DENV-infected cells in a contact-dependent manner. Once activated, they are the predominant producers of type 1 interferons (Navarro-Sánchez et al., 2005; Webster et al., 2016; Webster et al., 2018; Assil et al., 2019). Though the roles of pDCs in viral immunopathology is complex (Swiecki and Colonna, 2015), contraction of the pDC compartment in peripheral blood was associated with a higher risk of severe dengue in children (Rothman et al., 2003) suggesting that pDCs are critical to the successful control of dengue virus.

### NK Cells Are Activated and Recruited to Tissues During Infection

NK cells are bone marrow-derived innate immune lymphocytes that can kill virally infected cells, tumour cells, and antibody-opsonized cells/pathogens through a mechanism termed antibody-dependent cellular cytotoxicity (ADCC). Tissue resident cells including moDCs, macrophages [reviewed in (Michel et al., 2012)] and mast cells [reviewed in (Portales-Cervantes et al., 2019)] activated by initial infection (**Figure 1**, Step 2) and during the first “wave” rapidly recruit NK cells into infected organs and tissues in response to both DAMPS and viral infection, by secreting chemokines and cytokines (**Figure 1**, Step 3). Recruitment involves selective chemokine production in the microenviroment and corresponding receptor expression on NK cells. Several chemokines have been shown to mediate chemotaxis of NK cells to tissues including those typically known to direct chemotaxis and activation of monocytes [reviewed in (Shi and Pamer, 2011)] and mast cells [reviewed in (Halova et al., 2012)]. These include CCL2, CCL3, CCL4 (Morrison et al., 2003), CXCL2 (Burke et al., 2008), CXCL12 (Bernardini et al., 2008), and IFN (Paolini et al., 2015). Several chemokine receptors have been shown to mediate recruitment into infected tissues including CCR5, CXCR3, (Morrison et al., 2003; Huang et al., 2006), and sphingosine 1-phosphate receptor (Walzer et al., 2007), further expanding the chemokine ligands that can recruit NK cells.

Activation of NK-cell function is achieved by two distinct mechanisms: integration of signaling through a variety of inhibitory and activation receptors present on both the NK cells themselves and on host cells (Yokoyama et al., 2004; Jonsson and Yokoyama, 2009) and cytokine stimulation (Biron et al., 1999; Andrews et al., 2003; Cooper and Caligiuri, 2004). NK cell killing occurs by resting NK cells but is enhanced in response to cytokine stimulation. Typically, NK cells recognize “missing self” on infected cells when the host cell exhibits a down regulation in surface MHC class I molecules (Karre, 1995; Gasser and Raulet, 2006). NK cell killing activity is augmented by cytokine stimulation, including IL-12, IL-15, IL-18, and IL-21 (Cooper et al., 2001; Zwirner and Domaica, 2010; Brandstadter and Yang, 2011; Boieri et al., 2017) whereby cytokine-activated NK cells display enhanced cytotoxic activities (Henney et al., 1981; Boieri et al., 2017) and *de novo* cytokine synthesis that effectively amplifies the local immune response. Limiting infection spread early on is key to a rapid, effective, resolution and clearance of pathogen. NK are key effectors in controlling viral infections [reviewed in (Jost and Altfeld, 2013)] and are activated during dengue virus infection. In skin, St. John and colleagues, demonstrated early NK cell recruitment and activation at the site of DENV 2 inoculation in mouse footpads (St John et al., 2011). NK cell recruitment was dependent on mast cell activation and underscored how the innate immune system works in concert to mount an effective defense. More recently, human skin studies in DENV infected patients demonstrated CD69+CLA+ CXCR3+ CCR5+CD56 +NK cell recruitment to the skin during acute infection. These cells expressed proliferation markers and were increased during the febrile stage of illness, declining post-febrile, and in convalescence (Zimmer et al., 2019).

In several human studies circulating NK cells were found to express an activated phenotype with enhanced expression of CD69, a type II C‐lectin receptor and marker of lymphocyte activation. Homchampa et al. found evidence of NK cell cytotoxicity in children with acute dengue that correlated to disease severity (Homchampa et al., 1988) and later increased frequencies of circulating activated CD56+ CD69+ NK cells was observed in pediatric patients from Thailand with severe dengue disease as compared to patients with milder disease (Chen R. et al., 1999). In subsequent work, plasma from convalescent patients obtained after primary infection was found to mediate ADCC an *in vitro* NK cell killing assay (Laoprasopwattana et al., 2007) suggesting in the context of dengue, ADCC is likely also occurring.

An analysis of DENV patients from Brazil found an increase in circulating CD56+ NK cells during the acute phase of disease, defined as days 1–5 after onset of symptoms, with the majority of NK cells displaying early markers for activation including CD69, HLA-DR, and CD38, and increased expression of cytotoxic granule, TIA-1 (Azeredo et al., 2006) as compared to the late acute (days 6–10), or convalescence (days >11) patients. The group also showed that NK cell activating cytokine IL-15 was elevated in a significant number of patients during early acute infection (Azeredo et al., 2006).

## Neutrophils

Neutrophils are phagocytic granulocytes that are primarily involved in control of bacteria. Neutrophils are activated by DAMPs, PAMPs and pro-inflammatory cytokines and complement split products, including CXCL2, TNF, C5a, and C3a [reviewed in (Silvestre-Roig et al., 2016)]. The roles of neutrophils during viral infections, and in relation to outcome, are not well understood. One of the most potent outcomes of neutrophil activation is the release of NETs. NET formation (NETosis) is characterized by nuclear decondensation and delobulation, rupture of the plasma membrane, and release of DNA fibers with antimicrobial peptides and histones (Brinkmann et al., 2004) NETosis is a potent anti-microbial mechanism, but excessive formation of NETs, or the inability to clear NETs from the circulation, contributes to pathogenesis of both autoimmune diseases (Kaplan and Radic, 2012; Knight et al., 2012) and exacerbation of several different viral infections. Excessive NET formation results in exacerbated allergic airway inflammation during rhinovirus (RV) infection (Toussaint et al., 2017) and airway obstruction during respiratory syncytial virus (RSV) infection (Cortjens et al., 2016). Influenza virus infection also induces NETs in lungs of infected mice, though inhibition of NET formation did not affect infection outcomes (Hemmers et al., 2011). Together, the emerging data suggest that a neutrophil response to a viral infection may be more detrimental than beneficial, though more research is needed.

Recent work has demonstrated that neutrophils are activated during DENV infection. Studies in Vietnamese children with severe dengue have demonstrated neutrophil activation at the transcriptomic level (Hoang et al., 2010). DENV-infected patients display an increased number of circulating neutrophils during infection, suggesting that they are being activated (Thein et al., 2014 #37)]. More recently, neutrophil elastase activity (a key component of neutrophil granules) was increased DENV-infected patients, as compared to healthy controls, and levels were associated with severity of disease (Kunder et al., 2018). These data suggest that neutrophils may play an unrecognized role in dengue disease. To fully understand any relationship more work is needed.

## DENV: Escape From Skin and Replication in Lymph Nodes Leads to Development of Viremia

DENV escape is marked by migration of Langerhans cells and conventional dendritic cells stimulated by IL-1β and TNF (Stoitzner et al., 1999) out of the skin towards draining lymph nodes (Wu et al., 2000; Navarro-Sánchez et al., 2005; Cerny et al., 2014; Schmid and Harris, 2014; Duangkhae et al., 2018) (**Figure 1**, Step 4). Though some commentators presume that the mosquito feeding event results in viral particles being introduced directly into the bloodstream (Martina et al., 2009), this rarely, if ever, leads directly to viremia. Analysis of data from humans infected with DENV by mosquito bite has established a median intrinsic incubation period of 5.9 days, with 95% of subjects developing viremia between days 3 and 10 (Chan and Johansson, 2012). Therefore, it is much more likely that detection of DENV in the peripheral blood is a consequence of the egress of infected cells from the skin. This migration of infected phagocytes into regional lymph nodes 1) initiates the adaptive immune response, 2) precipitates further DENV replication in lymph node-resident and recruited mononuclear phagocytes, and 3) allows infectious DENV virions into the peripheral blood and the monocyte compartment (Castillo et al., 2018).

## Peripheral Blood—Monocytes

There is some disagreement as to which circulating cell type comprises the majority of DENV-infected cells. Early work using flow cytometry and immunocytochemistry analysis of blood from acutely ill dengue patients claimed that B cells were the main mononuclear cell fraction containing DENV (King et al., 1999). Later reports directly contradicted that, claiming that monocytes were the predominant infected cell type (Durbin et al., 2008; Schmid et al., 2014). More recently still, virus-inclusive single-cell RNA seq of peripheral blood from a limited number of dengue-infected patients showed that the majority of cells containing DENV RNA were B lymphocytes (Zanini et al., 2018). Importantly, the authors noted that the gene expression profiles of the sequenced B cells suggested that the virus may not be actively replicating. Despite perhaps not being the most numerous DENV-infected circulating cell type, monocytes are critical cellular players in dengue pathogenesis, Monocytes are the main target for DENV replication in peripheral blood (Halstead et al., 1977; Chen Y.-C. et al., 1999; Durbin et al., 2008; Zanini et al., 2018), primarily entering *via* the mannose receptor [reviewed in (Reyes-del Valle et al., 2014)].

Infection of monocytes occurs at a higher frequency in secondary dengue infection as a result of ADE. Functionally, ADE results in an increased proportion of infected monocytes, at least a 14-fold increase in *in vitro* experiments with DENV-1; ADE in this model also resulted in an increase in TNF secretion (Halstead and O’Rourke, 1977b). Infection of peripheral monocytes activated endothelial cells in a TNF dependent manner (Anderson et al., 1997), suggesting increased monocyte infection may lead to enhanced levels of TNF, driving more severe clinical disease.

## Distant Sites-Tissue Macrophages

Following dissemination of DENV from the skin to the peripheral blood (**Figure 1**, Step 5), and other organs in the body, other populations of specific tissue-resident macrophages are infected with DENV in the course of disease. These include Kupffer cells of the liver (Aye et al., 2014), and macrophages in the spleen (Balsitis et al., 2009). The infection of Kupffer cells and the resultant acute inflammation of the immediate area likely contributes to the observed elevation in liver enzymes ALT and AST (Fernando et al., 2016).

## The Adaptive Immune Response

The adaptive immune response is composed of both a humoral and a cell mediated component and is absolutely essential for controlling viral pathogens. Dengue is known to activate B cells and results in the production of virus-specific IgM, IgG, and IgA antibodies, a portion of which bind the viral envelope protein and neutralize virions, thereby preventing entry into target cells. IgE is produced during dengue infection, and as noted in the mast cell section, would serve to activate innate immune cells through the high affinity Fc epsilon receptor one expressed at high levels on mast cells and upregulated on activated dendritic cells. Importantly, prior infection and antibody levels are a major risk factor for development of more severe disease as discussed in the *Introduction*: (dengue virus and clinical spectra). In the case of a heterotypic infection with a different dengue serotype, patients are at increased risk to develop dengue hemorrhagic fever and/or dengue shock syndrome. In these cases, sub-neutralizing levels of cross reactive antibodies facilitate entry into increased numbers of target cells *via* Fc receptor mediated uptake (Halstead, 1979; Halstead, 1988; Halstead, 1994). This is thought to increase the overall infection burden, leading to higher titers of circulating virus and greater inflammation. The cell-mediated arm of the adaptive system consists of CD4+ helper T cells and CD8+ cytotoxic T cells that function to promote B cell activation and killing of virally infected host cells. CD8+ and CD4+ T cells are known to be activated in large numbers during dengue virus infection (Tian et al., 2019). Several studies have demonstrated that T cell epitopes are present across the viral proteome and T cell activation can result in both protective and pathogenic outcomes. Significant evidence suggests that dengue can induce cross reactive T cell activation, termed T cell original sin (Mongkolsapaya et al., 2003; Rothman, 2011). In this setting cross-reactive T cells, specific for the primary infecting serotype, become predominant during a secondary heterologous infection. This expansion of preexisting cross-reactive and low-affinity memory T cells is thought to hamper effective viral control and contribute to severe disease through enhanced production of inflammatory cytokines. However several studies have also demonstrated a protective role for dengue specific T cells in controlling infection. In murine models both CD4+ T cells and CD8+ T cells play a protective role against DENV infection preventing severe disease and facilitating viral clearance (Yauch et al., 2009; Zellweger et al., 2010; Zellweger et al., 2014; Ngono and Shresta, 2018). The protective role of T cells during dengue infections is underscored by studies both in murine model and in humans, that identified protective HLA alleles that are associated with strong and multifunctional T cell responses (Stephens et al., 2002; Stephens, 2010).

## Discussion

It is thought that the DENVs evolved into four distinct serotypes approximately 1,000 years ago and each of these four serotypes emerged into a cycle of transmission between humans and its mosquito vector approximately 125 to 320 years ago (Holmes and Twiddy, 2003; Twiddy et al., 2003a). Phylogenetic analysis suggests that the DENVs are rapidly evolving with major clade replacements and genetic shifts occurring in populations endemic for DENV (Holmes, 2003; Twiddy et al., 2003b; Zhang et al., 2005; Holmes, 2006). Asia in particular in this century has been pivotal in the evolution of DENV as the location of the first cases of the more severe form of DENV infection, dengue hemorrhagic fever (DHF), which made its first appearance in the 1950’s first in the Philippines then in Thailand (Halstead et al., 1967). This event was a hallmark denoting a change in the severity and pathogenesis of DENV viral-host interactions. The current Asian genotypes of each serotype are considered more severe and result in more severe dengue illness than the American genotypes (Kochel et al., 2002). Evidence suggests that DENV circulation in Asia due to its population growth and urbanization, high vector burden, and high level of pre-existing flavivirus seroprevalence, has contributed to the increase in genetic diversity of DENV which is estimated as increasing at a factor between 14 and 20 in the last 30 years (Twiddy et al., 2003a). The overall picture of DENV evolution in Asia and now the Americas is the active transmission of viruses in individuals who are highly flavivirus antibody experienced causing evolutionary pressure on the virus to evolve to escape and utilize pre-existing flavivirus immunity. By its nature the current evolving DENVs are adept at escaping heterologous neutralizing antibody and using it as a means to attain high viral load levels and more severe disease through antibody-dependent enhancement. Furthermore, in addition to the need to escape pre-existing adaptive immunity from antibody, the DENVs have evolved unique means to escape the host innate immune response as discussed above. Much needs to be understood about dengue pathogenesis and the viral-host interactions that result in severe disease. Utilization of the human infection model, improved techniques to understand the host genetic and immune response, and additional prospective human studies will further our understanding with application towards developing better drugs and vaccines to treat and prevent DENV infection.

## Author Contributions

All authors listed have made a substantial, direct. and intellectual contribution to the manuscript, and approved it for publication.

## Conflict of Interest

The authors declare that the research was conducted in the absence of any commercial or financial relationships that could be construed as a potential conflict of interest.
